# Effects of Long-Term Ayahuasca Administration on Memory and Anxiety in Rats

**DOI:** 10.1371/journal.pone.0145840

**Published:** 2015-12-30

**Authors:** Vanessa Manchim Favaro, Maurício Yonamine, Juliana Carlota Kramer Soares, Maria Gabriela Menezes Oliveira

**Affiliations:** 1 Departamento de Psicobiologia, Universidade Federal de São Paulo – UNIFESP, São Paulo, SP, Brazil; 2 Faculdade de Ciências Farmacêuticas, Departamento de Análises Clínicas e Toxicológicas, Universidade de São Paulo – USP, São Paulo, SP, Brazil; University of Otago, NEW ZEALAND

## Abstract

Ayahuasca is a hallucinogenic beverage that combines the action of the 5-HT_2A/2C_ agonist N,N-dimethyltryptamine (DMT) from *Psychotria viridis* with the monoamine oxidase inhibitors (MAOIs) induced by beta-carbonyls from *Banisteriopsis caapi*. Previous investigations have highlighted the involvement of ayahuasca with the activation of brain regions known to be involved with episodic memory, contextual associations and emotional processing after ayahuasca ingestion. Moreover long term users show better performance in neuropsychological tests when tested in off-drug condition. This study evaluated the effects of long-term administration of ayahuasca on Morris water maze (MWM), fear conditioning and elevated plus maze (EPM) performance in rats. Behavior tests started 48h after the end of treatment. Freeze-dried ayahuasca doses of 120, 240 and 480 mg/kg were used, with water as the control. Long-term administration consisted of a daily oral dose for 30 days by gavage. The behavioral data indicated that long-term ayahuasca administration did not affect the performance of animals in MWM and EPM tasks. However the dose of 120 mg/kg increased the contextual conditioned fear response for both background and foreground fear conditioning. The tone conditioned response was not affected after long-term administration. In addition, the increase in the contextual fear response was maintained during the repeated sessions several weeks after training. Taken together, these data showed that long-term ayahuasca administration in rats can interfere with the contextual association of emotional events, which is in agreement with the fact that the beverage activates brain areas related to these processes.

## Introduction

The hallucinogenic beverage ayahuasca was originally used by indigenous communities who lived throughout the upper Amazon and the Andes for the purposes of healing, spiritual development and divination [1]. The incorporation of elements from Catholicism and African religions to the ayahuasca ritual led to the creation of contemporary syncretic religious movements. Since the 1980s, churches in which ayahuasca is prepared and consumed have been spreading to urban centers initially in South America and then in North America and in Europe [2] [3].

Ayahuasca tea is prepared by decoction, boiling the bark and stems of *Banisteriopsis caapi* together with various admixture plants. The admixture most commonly employed is the leaves from *Psychotria viridis*. The pharmacological properties of this tea are dependent on a synergistic interaction between the active alkaloids in the plants. *P*. *viridis* contains the psychoactive agent N,N-dimethyltryptamine (DMT), whereas *B*. *caapi* contains substantial amounts of beta-carbolines, including harmine, harmaline and tetrahydroharmine as the primary alkaloids [3]. DMT is a potent hallucinogenic alkaloid structurally related to the neurotransmitter serotonin and has similarities with psychedelics, such as lysergic acid and mescaline [4] [5]. Beta-carboline alkaloids inhibit the metabolic breakdown of DMT by visceral MAO, allowing its access to systemic circulation [3]. In the central nervous system, DMT acts as agonist of 5-HT_2A/2C_ receptors [4] [6].

Recently it has shown that the administration of the tea in rats changes the serotonin levels in the amygdala and hippocampus, brain regions known to be related to the emotional behavior and memory [7]. However, despite of acting directly on the serotoninergic receptors, it was also observed an increase on the dopamine and noradrenaline levels in amygdala of animals [7].

The impact of acute ayahuasca consumption on the activation of limbic structures was also observed in human neuroimaging studies. Oral ayahuasca administration in volunteers increased the cerebral blood flow in regions known to be involved with episodic memory, contextual associations and emotional processing, such as the parahippocampal gyrus, the anterior cingulate cortex and the amygdala [8] [9]. In addition, long-term ayahuasca users have reported that regular use of ayahuasca in a religious context improves memory, concentration and a sense of meaning in their lives [10] [11]. Neuropsychological investigation have found that long-term ayahuasca users had better performance than control subjects on tests of frontal function [12] and on the WHO-UCLA auditory verbal learning test [10]. More interesting, the tests were conducted in an off-drug state, as the volunteers were not using the tea at the time of evaluation. The fact that these studies did not access the cognitive status of the subjects before the first use of ayahuasca, it is not possible to preclude the possibility that the groups were different before starting the ritual use of the tea. In addition, long-term prospective studies in humans are difficult to be conducted.

Animals’ studies can complement human findings in evaluating some cognitive or behavioral impact of long-term consumption. To investigate the long-term effects of ayahuasca on memory and anxiety processes, we used well-established paradigms, commonly adopted in pre-clinical drug trials. The Morris water maze (MWM) is a behavior paradigm commonly used to access hippocampus-dependent memory, as previous studies have shown that lesions in the hippocampus cause impairments in the performance of animals in the task [13] [14]. Additionally, fear conditioning is a model of associative emotional learning [15] [16], and the elevated plus maze (EPM) is a largely used ethological anxiety model [17] [18].

Therefore, the aim of the present investigation was to evaluate the effect of long-term daily oral administration of ayahuasca on MWM, contextual and tone fear conditioning and EPM performance of rats in an off-drug condition (48h after the last administration).

## Materials and Methods

### Ethics Statement

All procedures were approved by the Ethics Committee of Universidade Federal de São Paulo (n° 0793/11). The experiments were performed in compliance with the recommendations of “Brazilian Guidelines for Care and Use of animals for Scientific and Educational Purposes” (CONCEA, National Council of Animal Testing Control, Brazil).

### Subjects

Male *Wistar* rats weighing 300-350g were provided by the Centro de Desenvolvimento de Modelos Experimentais para Medicina e Biologia (CEDEME) of the Universidade Federal de São Paulo. The animals were housed in group of 5 animals per cage in a room with constant temperature of 22±1°C, with a 12 hour light/dark cycle (lights on at 7:00 a.m.). Food and water were available *ad libitum*.

### Ayahuasca

The ayahuasca tea was derived from a religious institution that uses the tea as a sacrament in their rituals. The responsible member of the religious group signed a consent form after being informed regarding the design of the study. The quantitative analysis of the alkaloids in the ayahuasca tea was performed by a previously described gas chromatography method [19]. The alkaloid concentrations were as follows: DMT = 0.26mg/mL, harmine = 0.56mg/mL, harmaline = 0.17mg/mL and tetrahydroharmine = 0.44mg/mL. Initially, the ayahuasca tea was concentrated in evaporator rotary until the tea reached 10% of the initial volume. The freeze-dried extract was placed in a lyophilizer at 4atm for 48h and then stored at 4°C.

### Doses and groups

The following freeze-dried ayahuasca extract doses were utilized: 120, 240 and 480 mg/kg. All animals received the concentrated freeze-dried ayahuasca from the same batch of the tea. For behavioral experiments, the freeze-dried extract was diluted in water and administered orally by gavage. The control group received water by the same procedure. We used 6–12 animals per group.

### Apparatus

Morris water maze: A 200cm diameter and approximately 40cm deep black circular pool was filled with water at 23°C to a height of 25cm. A 10cm diameter platform was placed inside the pool and was submerged 2cm below the water level. The pool was divided into four quadrants, and the platform was placed in a fixed region in the center of one of the quadrants. Distinct visual cues were positioned on each wall of the room for orientation.

Elevated plus maze: The EPM consisted of two open arms (50cm × 10cm × 1cm) and two enclosed arms (50cm × 10cm × 40cm) arranged such that same types of arms were opposite to each other. The maze was elevated to a height of 50cm.

Conditioning chamber: The chamber (MED-Associates, Inc.) consisted of aluminum walls and a Plexiglas rear wall, ceiling, and hinged front door (30cm × 24cm × 21cm), was situated in sound-attenuating cabinets in an isolated room. The floor of the chamber consisted of 19 stainless steel rods (4mm diameter and 1.5cm apart) connected to a shock source during training and contextual fear conditioning test. The conditioning chamber was cleaned with 30% alcohol solution. A white cylindrical chamber (35cm diameter and 30cm height) covered with transparent acrylic was used for the context B test in Experiment 2. The floor was also made of white acrylic. In this test, the conditioning chamber was cleaned with 10% sodium hypochlorite solution to characterize each chamber with a different scent. A black triangle and a plastic white floor were put inside the chamber to create a modified context for the tone fear conditioning test in Experiments 1. For this test, the chamber was cleaned with 10% sodium hypochlorite solution.

### Experimental procedures

#### Experiment 1. Effect of long-term ayahuasca administration on elevated plus maze and fear conditioning background performance

Different groups of animals received the administration of a daily oral dose of water or ayahuasca (120, 240 or 480 mg/kg) for 30 days. Forty-eight hours after the last administration, the subjects were placed on the center of the EPM facing an open arm and allowed to move freely for 5 minutes in the absence of the experimenter while their behavior was video recorded. An entry was defined as all four limbs into an arm. The animals were placed individually in the previously described conditioning chamber 30 minutes after the EPM. Three minutes after placement in the chamber, the rats received five tone (80dB, 5kHz, 5s)–footshock (1s, 0.6mA) pairings (25s intertrial intervals) in delay paradigm. The rats remained in the chamber for one additional minute, after which the rats were removed and returned to their home cages. Contextual fear conditioning (CFC) was assessed by returning the rats to the same training context, namely, to the conditioning chamber 24h later, and by assessing freezing, which is defined as complete immobility of the animal with the absence of vibrissa movements and sniffing, for 300s. Approximately 24h later, tone fear conditioning (TFC) was assessed by placing the rats in a previously described modified chamber, providing a different context, and at the end of the third minute of exposure to the apparatus, five tones (80dB, 5kHz, 5s) were presented, with 25s intertrial intervals. No footshock was delivered. Freezing time was measured both before and after tone presentation. After all of the above-described procedures were completed, the animals were returned to their home cages.

#### Experiment 2. Effect of long-term ayahuasca administration on performance in elevated plus maze and fear conditioning foreground followed by repeated sessions

Different groups of rats received the administration of a daily oral dose of water or ayahuasca 120mg/kg for 30 days. Forty-eight hours after the last administration, the subjects were submitted to the EPM as previously described in Experiment 1. Thirty minutes after the procedure, the animals were placed individually in the conditioning chamber. Three minutes after placement in the chamber, 5 footshocks (1s, 0.6mA) were delivered, with 29s intertrial intervals. No tone was presented in this experiment. CFC (context A) was assessed by returning the rats to the conditioning chambers 24h later and assessing freezing during 300s. Twenty-four hours after the context test, the animals were placed in the previously described cylindrical chamber (novel context B), and freezing was assessed for 300s. Repeated sessions were performed 7, 14, 21, 28 and 35 days after training, submitting the animals to the conditioning chamber (context A) and recording the freezing time for 300s. After all of the above-described procedures were completed, the animals were returned to their home cages.

#### Experiment 3. Effect of long-term ayahuasca administration on Morris water maze performance

Four groups of the animals received the administration of a daily oral dose of water or ayahuasca (120, 240 or 480mg/kg) for 30 days. Forty-eight hours after the last administration, the subjects were trained on the MWM—spatial version. The animals received a training session with 4 trials over 5 consecutive days. The sequence of starting points was randomly defined each day. When the animal could not find the platform after 1 minute, then the animal was conducted by the experimenter to the platform and remained there for approximately 20s before being removed from the pool. The interval between trials was 30s. After the last training day, animals underwent a probe session without the platform for 1 minute of free swimming. The time spent in the target quadrant in the probe session was used to determine the spatial memory. All trials were recorded by a camera and calculated by Noldus EthoVision version 7.0 software (Leesburg, VA, USA).

### Statistical analysis

The data from the EPM and from CFC were analyzed using one-way ANOVA or Student’s t-test. The data from TFC were analyzed using two-way repeated measures ANOVA, with groups and moments (pre-CS and CS intervals) as factors. The data from the MWM were analyzed using two-way repeated measures ANOVA, with groups and days (1 to 5 days of training) as factors, and the data from the MWM probe session were analyzed using two-way ANOVA, with groups and quadrants (target, opposite, right and left) as factors. *Post hoc* comparisons were performed, as necessary, using Tukey’s test when values were considered significant (p≤0.05). Statistical analyses were performed using the Statistica 7.0 (Statsoft; Tulsa, OK).

## Results

### Experiment 1

**Fig 1:**
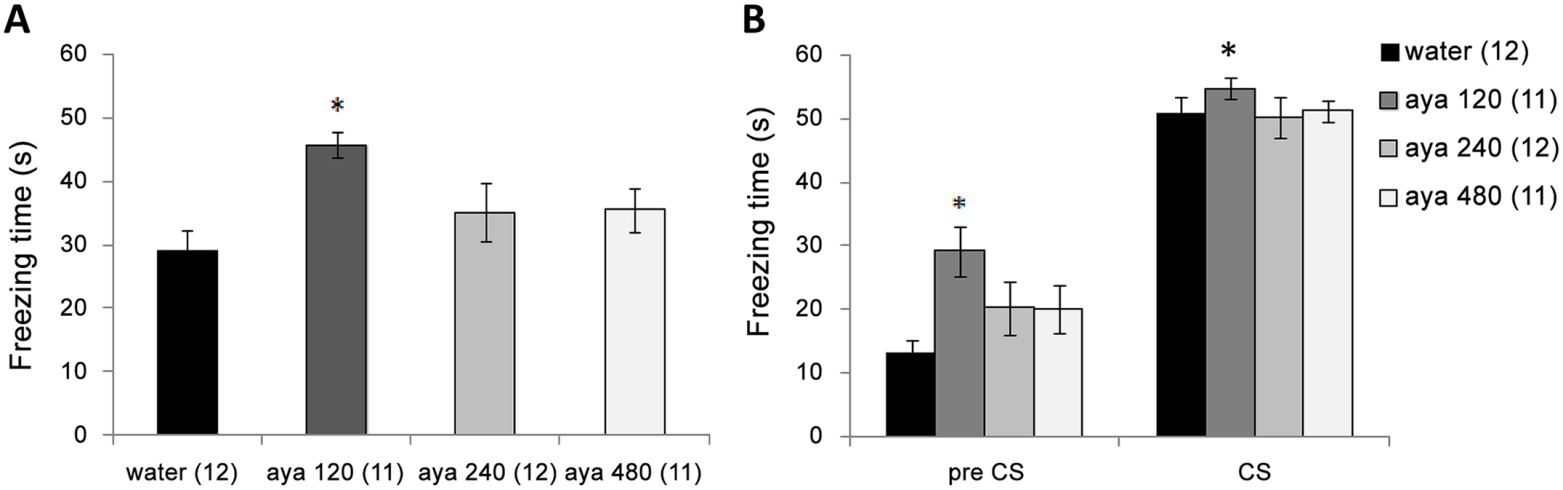
Effect of long-term ayahuasca administration on fear conditioning background performance. This figure shows the mean values (±SEM) of the freezing times during CFC (**A**) and TFC (**B**) tests of animals that received water or ayahuasca at doses of 120 mg/kg (aya 120), 240 mg/kg (aya 240) and 480 mg/kg (aya 480). “*” indicates a significant difference compared with the water group (p≤0.05). The number of animals per group is shown in parentheses after group names.

The one-way ANOVA results indicated a significant difference among the groups [F_(3,42)_ = 3.8; p = 0.01], in which the *post hoc* Tukey’s test demonstrated that animals that received the 120mg/kg ayahuasca dose had increased fear responses when compared with the control group in CFC test (Fig 1A). For the TFC test, the two-way repeated measures ANOVA results indicated a significant group effect [F_(3,42)_ = 2.76; p = 0.05] in which the *post hoc* Tukey’s test demonstrated that animals that received a 120mg/kg ayahuasca dose had increased fear responses when compared with the control group. We also observed a significant time effect [F_(1,42)_ = 324.8; p≤0.001] with freezing levels in the CS moment higher than pre-CS moment levels. The group × moment interactions were not significant [F_(3,42)_ = 2.05; p = 0.12] (Fig 1B). The anxiety results are showed in the Table 1. In short, the one-way ANOVA showed no difference among groups in any parameters investigated.

**Table 1:** Effect of ayahuasca administration on performance in elevated plus maze.

Experiment	Group	% Open arm time spent	% Open arm entries	Frequency of rearing	Frequency of head dip
Exp 1	One-way ANOVA	F_(3,42)_ = 0.54;P = 0.65	F_(3,42)_ = 0.50;P = 0.68	F_(3,42)_ = 0.74;P = 0.53	F_(3,42)_ = 0.05;P = 0.98
	Water (12)	20 ± 3	34 ± 2	15 ± 2	10 ± 1
	Aya 120 (11)	21 ± 2	33 ± 3	12 ± 1	10 ± 1
	Aya 240 (12)	20 ± 3	32 ± 4	13 ± 2	10 ± 1
	Aya 480 (11)	16 ± 2	32 ± 3	13 ± 1	9 ± 1
Exp 2	Student′s t-test	t_(21)_ = 0.87;P = 0.39	t_(21)_ = -0.55;P = 0.58	t_(21)_ = -0.47;P = 0.64	t_(21)_ = 0.32;P = 0.74
	Water (12)	23 ± 3	37 ± 3	12 ± 1	9 ± 1
	Aya 120 (12)	19 ± 3	39 ± 3	13 ± 1	9 ± 1

Note: Data are expressed as mean ± SEM. The number of animals per group is shown in parentheses after the group names.

### Experiment 2

**Fig 2:**
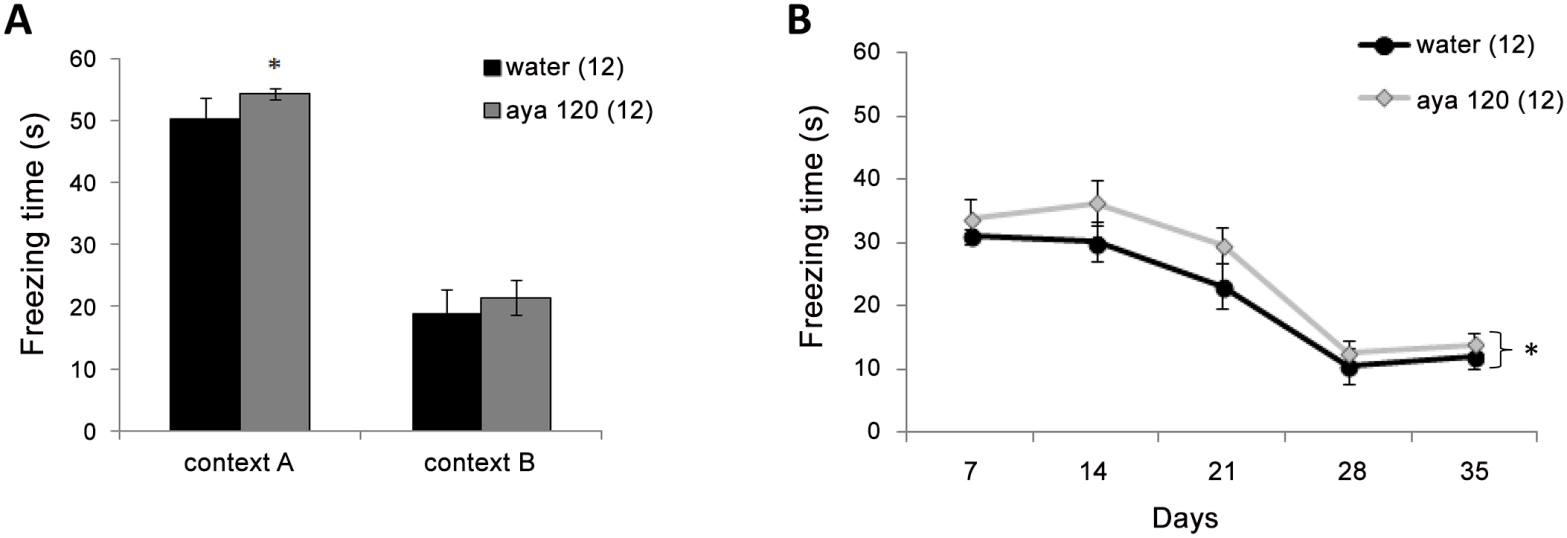
The effect of long-term ayahuasca administration on fear conditioning foreground followed by repeated sessions. This figure shows the mean values (±SEM) of the freezing times during context A and B tests (**A**) and during the repeated sessions 7, 14, 21, 28 and 35 days after training (**B**) of the animals that received water or ayahuasca at a dose of 120 mg/kg (aya 120). “*” indicates a significant difference compared with the water group (p≤0.05). The number of animals per group is shown in parentheses after group names.

For the foreground fear conditioning, the Student’s t-test results indicated that the group that received a 120 mg/kg ayahuasca dose had an increased freezing time compared with the control group in context A [t_(22)_ = -2.32; p = 0.03]; the same effect was not observed in context B [t_(22)_ = -1.09; p = 0.28] (Fig 2A). For the repeated sessions, the two-way repeated measures ANOVA results indicated no group × time interactions [F_(4,88)_ = 1.91; p = 0.11] but a significant group effect [F_(1,22)_ = 9.4; p≤0.001] and time effect [F_(4,88)_ = 34.4; p≤0.001], in which Tukey’s test showed that freezing levels decreased across sessions. (Fig 2B). The anxiety results are showed in the Table 1. In short, the one-way ANOVA test showed no difference between groups in any parameters investigated.

### Experiment 3

The two-way repeated measures ANOVA followed by Tukey’s *post hoc* test showed a decreasing in latencies through training period indicating that all groups learned the task F_(4,368)_ = 59.9; p≤0.001]. Moreover no group effects [F_(3,92)_ = 0.67; p = 0.57] or group × training period interactions [F_(12,368)_ = 1.11; p = 0.54] were observed in the MWM training. For the MWM probe test, all groups spent more time in the target quadrant (Table 2) confirming learning of the task and no difference among groups was observed.

**Table 2:** Effect of long-term ayahuasca administration on Morris water maze performance.

	Probe test—Time spent in quadrants (s)			
Group	Target	Opposite	Right	Left
Water (6)	29 ± 8*	11 ± 3	13 ± 6	7 ± 2
Aya 120 (6)	26 ± 7*	13 ± 3	14 ± 5	8 ± 2
Aya 240 (6)	27 ± 6*	12 ± 4	15 ± 5	6 ± 3
Aya 480 (6)	26 ± 9*	11 ± 3	14 ± 6	8 ± 3

Note: Data are expressed as mean ± SEM. The number of animals per group is shown in parentheses after the group names.

* Means different from opposite, right and left quadrants (p≤0.05). The two-way ANOVA results indicated a significant quadrant effect [F_(3,60)_ = 110.96; p≤0.001], in which Tukey’s test indicated that the time spent in the target differed from the other quadrants. The group effects [F_(3,20)_ = 0.68; p = 0.57] and the group × quadrant interactions [F_(9,60)_ = 0.51; p = 0.85] were not significant.

## Discussion

The aim of the present study was to investigate whether long-term ayahuasca administration influences the behavior of rats in elevated plus maze, fear conditioning and Morris water maze paradigms. To our knowledge, this study is the first to associate a long-term ayahuasca treatment with these experimental models in rodents. In short, daily oral treatment did not affect the MWM. However, one of the doses used, lead to an increased CFC without increasing TFC or anxiety-like behavior as evaluated by the EPM. It was observed some generalized contextual fear response, but only when the test context had common physical characteristics that resembled the training context. It is important to note that the behavior changes observed were acquired in off-drug state as the training was conducted 48 hours after the last dose administered.

The concentration of ayahuasca components obtained by quantitative analysis in gas chromatography was similar to those values found in the literature [19] [20]. The temperature during the concentration process was controlled to avoid the evaporation of the primary alkaloids. The primary alkaloids are responsible for producing psychoactive effects, which range from perceptual changes to emotional and cognitive changes, with primary action on the serotonergic system. All of the doses utilizing the freeze-dried extract were achieved considering the animal weight and the DMT concentration in the ayahuasca tea consumed during the religious ceremony [20].

The lower dose used in the present study had an impact on the performance of the animals in the fear conditioning test. The animals that received the 120 mg/kg ayahuasca dose for 30 days showed increased freezing levels compared with the control group in both background and foreground contextual fear conditioning (Experiments 1 and 2). Foreground fear conditioning differs from the background where the auditory CS is presented with the context. The context is more salient in the foreground fear conditioning. So, the results of both experiments show that the nature of context being salient or not during the learning and memory process is affected by long-term ayahuasca administration. For both types of learning the hippocampus and the amygdala are relevant structures for contextual association in the fear conditioning paradigm [21] [22] [16]. Moreover, the single ayahuasca administration in rats leads to an increased release of inhibitory amino acids in the hippocampus and an increased level of monoamines in the amygdala after different doses [7]. Evidences from human studies shows that ayahuasca intake activates brain regions related to emotional learning and memory processes such as the parahippocampal gyrus, the anterior cingulate cortex and the amygdala [7] [8]. Although our study has not been designed to investigate directly any brain structures, the results are consistent with human and animal literature.

The same dose (120mg/kg) that enhanced contextual fear conditioning increased the fear response in the TFC test for both before and after tone presentation (Experiment 1). This effect can be not interpreted as in increased tone fear conditioning response. TFC increasing would be considered if a higher freezing response was observed only after the tone presentation, and not before. One possible explanation for the pre-tone increased freezing is that the treatment could induce an increased sensitization to the shock experience that could be expressed as an enhanced response to the new environment. Alternatively, the pre-tone increased freezing could be due to fact that the treated rat is generalizing the elements of the training chamber. This possibility was raised by the fact that in Experiment 1, the training and testing contexts had some common elements, such as the grid floor and one of the walls of the used chamber. The second experiment was design to test this hypothesis. When the animals were exposed to a test chamber completely differed from the training chamber the behavior of the treated animals did not differ from the control group in tone fear conditioning. Therefore, the higher freezing performed in the pre-CS moment during TFC (Experiment 1) was due to similarities between contexts, leading to a conditioned response to the common contextual elements for the 120 mg/kg ayahuasca group. Repeated exposure to the context of training without the aversive stimulus lead to the extinction of the conditioned response in both groups. However the ayahuasca group maintained the initial difference from the control group (Experiment 2).

A control experiment was conducted to investigate whether the effect observed in the fear conditioning was a consequence of the long-term ayahuasca administration or could be due to a residual effect of the last single dose administered 48h before training. The data showed that the single ayahuasca administration given 48 hours before the beginning of the task was insufficient to cause any behavior changes in the fear conditioning (data not shown). Taken together, the results of these experiments suggest that the increased learning observed in Experiments 1 and 2 is possibly related to the consequences of the long-term ayahuasca administration.

To evaluate if the long-term ayahuasca administration also interferes with spatial learning, MWM was conducted with the animals in an off-drug state. According to the results of the Experiment 3, all animals were able to learn the task. Moreover long-term ayahuasca administration did not induce any significant behavior changes in the performance of animals in the probe session of the MWM. These results suggest that long-term ayahuasca administration did not change the spatial learning and memory processes involved in solving the MWM task in all doses used. The same treatment and dose that improved CFC had no effect on a task usually used investigate hippocampal dependent learning that do not involve classical conditioning. It is possible to interpret that the long-term ayahuasca administration may interfere with the contextual association with emotional events which only occurs in the CFC.

Ayahuasca tea drinkers have reported remission of anxiety disorders after beginning to drink the tea in the religious context [10]. To verify the possibility of an anxiety effect after a long-term treatment, EPM were conducted in the Experiments 1 and 2. We found no evidence of long-term ayahuasca administration influence on EPM performance in all experiments. In accordance with our results, Santos et. al. (2007) shows no anxiety alteration after ayahuasca consumption at long-term drinkers measured by the state–trait anxiety inventory (STAI). One can argue that the anxiolytic effect observed in Grob et al. (1996) study could be related more to the religious context than to the pharmacological effect of ayahuasca, or possibly to the combination of both. Unfortunately, the studies linking religiosity and anxiety show contradictory results as assigning increased or decreased anxiety to religiosity and even no religiosity-anxiety relation [23]. The attempt to examine the relation between religiosity and anxiety is difficult due to methodological and conceptual problems that can contribute to the contradictory findings [23].

The present study has some limitations. First, we adopted the administration for 30 days to maximize the possibility of observing altered behaviors. Any negative data with a less massive schedule would be subject to the criticism that the treatment was insufficient to induce altered behaviors. Second, the interval between the last day of treatment and the first day of training were given to observe the off-drug state effect of ayahuasca and not its influence during all of the behavior tasks. Both schemes of protocol are not equivalent to the usual frequency of ayahuasca intake of experienced drinkers.

Taken together, these data suggest that long-term ayahuasca administration in rats may interfere with the contextual association with emotional events necessary for CFC. They provide clear evidences to the possibility of the long-term treatment lead to some sort of biological plastic alteration that result in an improvement of this specific kind of learning (contextual conditioning). In other words, the data obtained in the present study show the possibility of modifications in specifics learning processes evaluated through animal behavior analysis even after the end of treatment. This knowledge is essential for a better understanding of long-term ayahuasca effects and may guide further studies regarding the role of ayahuasca in related memory and anxiety behaviors. Moreover, although the behavioral observations made in rats cannot be directly extrapolated to humans, our results highlight the importance to include this type of emotional memory testing in humans.
